# Industry-level emotional-labor exposure proxy and employee well-being: the statistical indirect role of workplace equity perceptions

**DOI:** 10.3389/fpsyg.2026.1902210

**Published:** 2026-09-18

**Authors:** Yaohua Liao, Lirong Wen, Junming Yang

**Affiliations:** Modern School of Management, Zhejiang Industry and Trade Vocational College, Wenzhou, Zhejiang, China

**Keywords:** China labor force, CLDS 2018, emotional labor, employee well-being, industry-level exposure proxy, occupational health psychology, organizational justice, statistical indirect association

## Abstract

**Background:**

Emotional labor is often examined as an individual emotion-regulation strategy, but national labor-force data can also capture the industry contexts in which emotional demands are more likely to occur. This study examines whether workplace equity perceptions are statistically consistent with an indirect association between an industry-level emotional-labor exposure proxy (IELR) and employee well-being.

**Methods:**

The analysis used the CLDS 2018 Individual Questionnaire file that is short for CLDS 2018 IID.dta in this study from the China Labor-force Dynamics Survey(CLDS) by the Center for Social Science Survey at Sun Yat-sen University in Guangzhou, China, Which contains 16,537 records and 1,517 variables. All 50 variables used in the analysis script were available. After retaining working respondents with industry data and applying a common complete analytical sample rule with at least three of five workplace-equity items, the main sample included 9,361 respondents. The paid-employee robustness sample included 1,963 respondents after native missing values for labor-contract and overtime variables were preserved. IELR was coded as a five-level industry exposure proxy, not as an individual emotional-labor scale. Province fixed effects were included in the reported models, and bootstrap confidence intervals were calculated for both the main and paid-employee samples.

**Results:**

In the main sample, IELR was positively associated with workplace equity perceptions (β = 0.063, *p* < 0.001) and showed a small positive direct association with well-being after equity was included (β = 0.038, *p* < 0.001). Workplace equity was a much stronger correlate of well-being (β = 0.460, *p* < 0.001). The main indirect association via equity was 0.029, with a 95% bootstrap confidence interval of 0.019 to 0.039. In the paid-employee sample, the direct IELR-well-being association was not statistically detectable (β = 0.016, *p* = 0.463), and the indirect association was small with a 95% bootstrap interval crossing zero.

**Conclusion:**

Workplace equity is the more stable and substantively important correlate of well-being. IELR should be interpreted only as an exploratory industry-level exposure proxy, and all findings remain associational because the dataset is cross-sectional.

## Introduction

Emotional aspects of work are done in a hidden way. The concept of emotional labor was initially defined as the regulation of emotions in employment tasks, particularly when they involve the management of feeling and publicly observable emotional display in service encounters (Hochschild, 1983). Subsequent research in the field of organizational research found that emotional labor could be broken down into four elements: frequency of emotional display, attention to display rules, emotional variety, and emotional dissonance (Morris and Feldman, 1996), and that emotional-labor strategies could be divided into three categories: surface acting, deep acting, and naturally felt emotion (Diefendorff et al., 2005). There is a need for careful handling of emotional labor in this study, as CLDS 2018 is unable to measure these individual strategies directly, but can provide an industry-level exposure proxy for the kinds of industries where emotional demands are more likely to be public-facing. Recent studies provide related empirical evidence at the empirical level: daily emotional labor, hospitality and tourism evidence, and nursing reviews connect emotional demands with health, burnout, and service outcomes, while service and leadership studies show that emotional labor varies with work roles and relational demands (Cheng et al., 2025; Cheng and Zhao, 2025; Feng et al., 2024; Lee et al., 2021; Riforgiate et al., 2022; Xu et al., 2020).

In this context, work design and industry structure are associated with emotions. Consequences for not following the rules of interpersonal display vary by sector, rules shown in interpersonal interactions vary by role, and frequency of interpersonal encounters varies by role. The present study thus conceptualizes industry as an exposure context instead of a context where all workers engage in the same form of emotional labor. It can be used when secondary national data sources include industry and well-being indicators, but do not include validated scales for emotional labor. The second topic reviews an applied policy question: What are the conditions at work that correlate with improved employee well-being in the case in which the task is too emotional to change completely?

One of these is workplace equity. Equity is not solely about equal pay; it is also about how people think about the opportunities and rewards they have to learn, grow and develop; how they are treated; and whether they are listened to. One of these is emotional labor, when employees are expected to regulate their emotions for customers, patients, students, clients or the public, they might be especially sensitive to whether or not the organisation values them. Emotional needs can arise when workers feel that they are devalued, ignored, disrespected or powerless, and they may feel as if their workload is not appreciated, but their job is not. Fair treatment, on the other hand, could instead be used to help employees to understand that emotion-intensive jobs are skilful, meaningful, and socially beneficial. This justice framing can also be looked back to in classic theories; distributive, procedural, and interpersonal theories of justice provide a theoretical basis for expecting that pay, promotion processes, respectful treatment, and voice can influence the evaluation of emotionally demanding work by employees (Colquitt, 2001; Greenberg, 1987).

A useful empirical context for this question can be provided by China. Emotional demands and perceived fairness are becoming more relevant in the labor force because of the fast growth of the service sector, urbanization and the diversification of both formal and informal work, as well as altered notions of work quality. The China Labor-force Dynamics Survey (CLDS) is an appropriate dataset for this purpose as it includes data on work, industry, income, subjective well-being, health, depression symptoms and perceptions of fairness. The dataset allows, therefore, for an unobscured and replicable secondary analysis of the relationship between industry exposure and perceived equity and wellbeing on a large working sample. Deliberately, the study is not too ambitious in terms of causal language. The difference is that due to the secondary nature of this data, CLDS 2018 cannot be used to prove the causal effects of industry emotional-labor requirements on equity perceptions or well-being. Instead, associations between industry context and well-being are estimated conditional on equity perceptions, and an indirect pathway is estimated consistent with the theoretical argument that equity perceptions could explain why industry context is associated with well-being. This is a critical demarcation that emotional labor studies have at times suffered from making definite causal statements founded on one-wave survey data.

The study has four limited contributions. First, it connects classic emotional-labor theory with a cautious industry-level exposure proxy that can be used when national labor-force data lack individual emotional-labor scales. Second, it treats workplace equity as a general justice-related perception index and identifies how the five CLDS items correspond to distributive, procedural, and interpersonal justice. Third, it estimates province-fixed-effect association models and bootstrapped statistical indirect associations using the corrected CLDS 2018 analytical sample. Fourth, it reports paid-employee, cluster-standard-error, no-income, alternative well-being, and IELR-mapping checks so that the empirical claims remain transparent and bounded.

## Literature review and hypothesis development

### Emotional labor as an industry-level work demand

The notion of emotional labor started with the recognition that paid labor was not just physical or mental work, but emotional, as well (Hochschild, 1983). Then Morris and Feldman (1996) defined emotional labor as a role demand that was contingent on display rules and interactional intensity and Grandey (2000) framed it in emotion-regulation theory. Brotheridge and Lee (2003) developed and validated measures of emotional-labor demands and the surface- and deep-acting strategies. The reasons for these classic foundations are that the current study does not claim that it observes surface acting or deep acting. Rather, it looks into whether industry categories are suitable as a clear, exploratory exposure context for a secondary labor force dataset for potential emotional demands.

This distinction is relevant as CLDS 2018 does not include a personal, validated emotional-labor scale. This is because it is not an accurate measurement of individual emotion regulation. In the present study, therefore, industry is used as a proxy for context level, in line with the notion that occupations and industries are different in public contact, service interaction, care work and conflict management expectations. This logic is similar to the occupational information systems that describe features of a job in terms of tasks, work activities, work context, and interpersonal requirements, like O*NET, and grouping occupations into definitions by tasks and duties, like ISCO-08 (National Center for O*NET Development, n.d.; International Labour Office, 2012). Mapping used here remains exploratory and will need external validation using indicators of emotional demands coded into occupations in the future.

### Workplace equity, justice, and employee dignity

The idea of workplace equity is based on an organizational justice theory. Distributive justice (perceived fairness of outcomes), procedural justice (fairness of decision processes), interpersonal justice (respectful treatment), and informational justice (adequate explanation) are classic forms of justice (Colquitt, 2001; Greenberg, 1987). These are especially important for the dimensions of emotionally demanding work, as it is the nature of such work to require employees to respond to the emotions of customers, patients, students, clients or the public; they may be particularly attuned to how the organization treats that response. The general fairness measure includes an overall justice judgment, the income satisfaction measure reflects mostly distributive justice, the promotion satisfaction measure reflects both distributive and procedural justice, the respect measure reflects interpersonal justice, and the voice/opinion expression measure reflects procedural justice. Due to the limited number of relevant items available in CLDS 2018, the study does not measure individual justice subscales, but rather a general workplace-equity perception index. Recent reviews of organizational justice and health, together with employment-system approaches to equity, support treating fairness as a broad workplace resource rather than only a single pay issue (Cachón-Alonso and Elovainio, 2022; Lai et al., 2024).

Equity is also related to identity and social belonging. But if they feel important to the team (or the team is important to them) and their role is meaningful, then they will feel the importance of the team and the goals, and they will feel hard work as meaningful. Social identity theories, therefore, can explain the possibility of treating work fairly to make hard work shared work (not individual work) (Tyler and Blader, 2003). Equity is not the same in all places and at all times. Gender bias and discrimination exist and are manifested in constraints on access to respect, advancement and recognition in the workplace (Heilman et al., 2024). Equity in the workplace is also suggested as varying across various job systems such as pay, promotion opportunities, supervision, and resources provided to employees (Trejo et al., 2025).

### Workplace equity and well-being

The health of employees is complex. The subjective well-being tradition of Diener (1984) and the psychological well-being tradition of Ryff (1989) focus on different components of well-being. There is no measure in the CLDS 2018 that can replicate either of these frameworks completely. It is instead a secondary-data composite that combines life happiness, life satisfaction, economic satisfaction, positive self-rated health and having a low level of depressive symptoms. The life-evaluation items are most similar to Diener tradition items, and health and low symptoms give a restricted sense of functioning, non-distress, and a general sense of psychological well-being. This boundary is given because the index is a pragmatic measure of the labor force, rather than a designed scale of wellbeing. Broader well-being studies also show that positive leadership, ethical leadership, and cross-cultural work settings are relevant for employee well-being, which supports reading the CLDS composite as a practical labor-force outcome rather than a narrow clinical measure (Azila-Gbettor et al., 2024; Fu et al., 2020; Sarwar et al., 2020).

There are several empirical studies that have included justice/fairness in explanations of well-being. Aggarwal and Mittal (2021) identified that employee’s well-being is a consequence of employee engagement, which is related to organizational justice. Similarly, Magnavita et al. (2022) showed that organizational justice is correlated with health outcomes for hospital staff, indicating that organizational justice is not just a satisfaction issue but a health issue as well. Emotional laborers’ well-being is also affected by management quality. Wang (2020) showed that hotel employees’ emotional-labor strategies were associated with service quality, engagement in service groups of hotels. In nursing services, internal service quality was positively associated with job satisfaction, organizational commitment, employee well-being, and job performance (Abdullah et al., 2021).

### Emotional labor, equity, and the statistical indirect role of fairness perceptions

Theoretically, a mediation argument for this is that the emotional-labor requirements of the industry that employees join affect the type of workplace they will enter, and workplace equity perceptions directly impact an employee’s evaluation of their life and work. Kim and Leach (2021) showed that organizational justice acts as a buffer against customer-support workers’ burnout who have high levels of emotional labor in their jobs. In recent years, the evidence for nursing shows that perceived organizational justice is also related to emotional labor and downstream work behaviors (Meng et al., 2024).

Emotional demands are not necessarily eliminated in a fair workplace, but they might be altered. When emotion-intensive work is accompanied by fair compensation, respectful treatment, voice, and promotion opportunity, then the employee may feel emotion-intensive work is recognized labor. Leadership research links authentic leadership to trust and work engagement, and ethical leadership to employee well-being through emotion management (Baquero, 2023; Saleem et al., 2024). A broader body of literature related to organizational health also backs the link between equity and health. Workplace health-promotion practices were positively associated with employee well-being and loyalty during COVID-19 (Görgényi-Hegyes et al., 2021). Flexible and sustainable HR practices are also linked to working conditions and job satisfaction and performance (Davidescu et al., 2020).

Emotional labor is not always bad for wellbeing, it depends on the resources surrounding the job. Suh and Punnett (2021) demonstrated that their surface-acting emotional labor is a predictor of depressive symptoms across time in the healthcare profession. Similarly, Zou et al. (2022) demonstrated that the choice of emotional labor strategies to be used in the workplace can be a mechanism by which service employee well-being is supported by workplace spirituality. Work has been ongoing to link ethics, emotion and well-being in recent times. In their study, Saleem et al. (2024) discovered that the ethical leader behavior has a positive effect on employee well-being via emotion management. Ertemsir et al. (2024) found that workplace spirituality moderates the effect of organizational justice on job satisfaction, supporting the view that justice operates within a broader context of workplace meaning. Burnout research is another rationale for attention to fairness. Emotional labor, particularly when excessive or dissonant, was positively associated with job burnout (Chen et al., 2024). Wu et al. (2024) linked emotional labor to service quality through work fatigue, with supervisor-subordinate guanxi as a moderator. Santiago-Torner et al. (2024) examined ethical leadership and emotional exhaustion in virtual work through work autonomy and teleworking intensity. Thus, an equal emotional need can mean different things to health in a just and equitable workplace.

### Hypotheses and research questions

Based on this literature and the available CLDS 2018 measures, the study asks four research questions. RQ1: How are industry-level emotional-labor exposure proxy scores distributed across the working CLDS 2018 sample? RQ2: Is IELR associated with employee well-being after adjustment for demographic, income, workload, hukou, and province differences? RQ3: Are workplace equity perceptions associated with employee well-being, psychological distress, and work strain? RQ4: Are workplace equity perceptions statistically consistent with an indirect association between IELR and employee well-being?

*H1*: IELR is associated with employee well-being.

*H2*: IELR is associated with workplace equity perceptions.

*H3*: Workplace equity perceptions are positively associated with employee well-being.

*H4*: Workplace equity perceptions are statistically consistent with an indirect association between IELR and employee well-being.

*H5*: Workplace equity perceptions are negatively associated with psychological distress and work strain.

The full theoretical logic is summarized in Figure 1, which places workplace equity perceptions between industry emotional-labor requirement exposure and employee well-being while keeping the control variables analytically separate.

**Figure 1:**
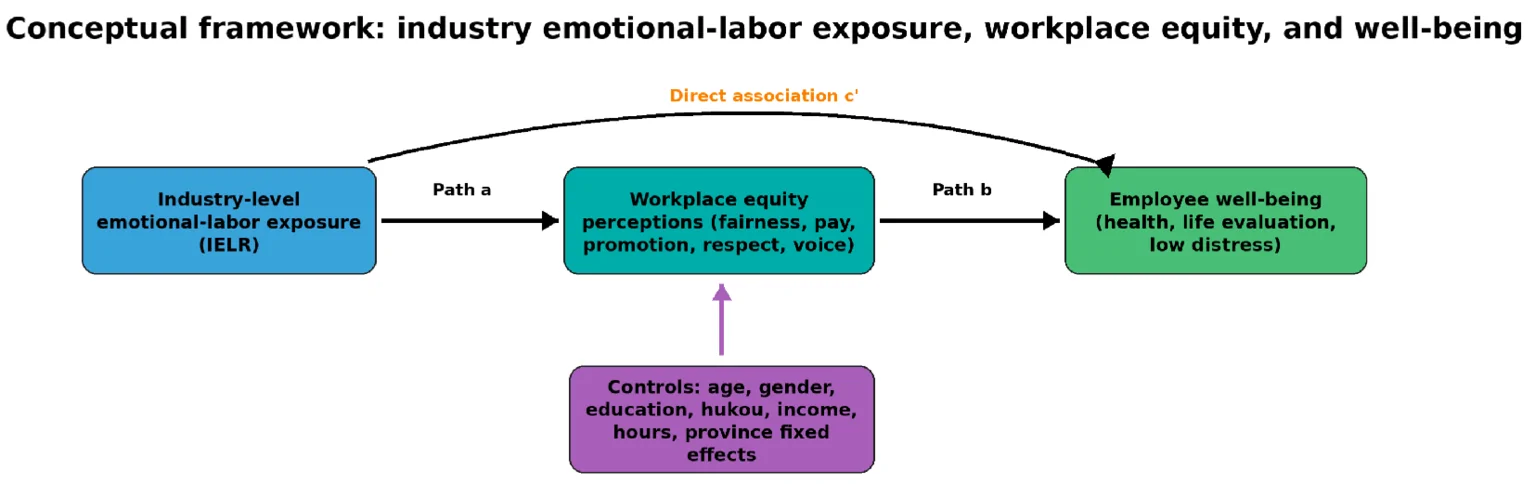
Conceptual framework linking industry emotional-labor requirement exposure, workplace equity perceptions, and employee well-being. IELR is an industry-level exposure proxy, not an individual emotional-labor scale.

Table 1 links each research question with the dataset variables and analysis used to answer it, ensuring that the title, methods, results and discussion remain aligned.

**Table 1:** Research questions and analytical correspondence.

RQ	Research question	Variables used	Analysis
RQ1	How are IELR scores distributed across the working sample?	Industry groups, IELR score, sample counts	Descriptive statistics and industry profile
RQ2	Is IELR associated with well-being?	IELR, well-being index, controls	Robust OLS regression
RQ3	Is workplace equity associated with well-being, distress, and strain?	Equity index, well-being, CES-D distress, work strain	Correlation and robust OLS regression
RQ4	Are workplace equity perceptions statistically consistent with an indirect association between IELR and well-being?	IELR, equity, well-being	Bootstrap statistical indirect-association analysis

Each research question is answerable using variables available in the CLDS 2018 analytical dataset.

## Materials and methods

### Research design

This study adopted a quantitative, cross-sectional, secondary-data design. The analysis is regression-based and estimates statistical associations rather than experimental or causal effects. Because the data are cross-sectional, the indirect-association models are interpreted as statistical patterns consistent with the proposed framework, not as evidence of temporal ordering.

### Data source

The analysis used the CLDS2018 IID.dta from the China Labor-force Dynamics Survey (CLDS) by the Center for Social Science Survey at Sun Yat-sen University in Guangzhou, China. The raw file contains 16,537 records and 1,517 variables. All 50 variables invoked by the final analysis script were present in the dataset, and the individual identifier contained no missing or duplicate values.

The data set is relevant to the study aim because it contains the industry categories, work-related perceptions, well-being indicators, items of depressive symptoms, work strain indicators, and demographic controls. It does not contain a direct emotional-labor scale; therefore, the operationalization of the emotional-labor requirements was done transparently at industry level, instead of self-reported emotional-labor behavior on a single scale.

### Sample construction

The analytical sample was built in stages. First, all 16,537 records in IID.dta were inspected. Second, 10,648 working respondents with usable industry information were retained. Third, analytical variables were cleaned using variable-specific validity checks rather than a single global missing-code rule. Fourth, the main sample was defined as a common complete analytical sample for the main and supplementary outcome models, requiring valid exposure, workplace-equity, well-being, psychological-distress, work-strain, and control variables, with at least three valid workplace-equity items out of five. This produced a main analytical sample of 9,361 respondents, representing 87.9% of the 10,648 working respondents with industry data. The paid-employee robustness sample retained 2,348 paid employees with industry data and 1,963 complete cases after labor-contract and overtime missing values were preserved.

### Measures

Industry emotional-labor requirement exposure was coded as a five-level industry-sector score. The coding followed a pre-specified rubric based on expected public contact, customer or client service, care-oriented interaction, interpersonal display requirements, and conflict-management demands. This logic is conceptually consistent with how occupational-information systems such as O*NET describe jobs through work activities, task content, work context, and interpersonal work requirements, and with ISCO-08’s task-and-duty basis for grouping occupations (International Labour Office, 2012; National Center for O*NET Development, n.d.). However, the CLDS industry code is broader than an occupation code and does not provide direct emotional-demand ratings. The measure is therefore an exploratory industry-level exposure proxy and should not be interpreted as an individual emotional-labor scale. The full industry-to-score mapping is provided in Supplementary Table S1 and is implemented directly in the accompanying Python script without using integer-key **kwargs syntax. The IELR coding logic aligns with O*NET and ISCO-08 occupational-classification principles because those frameworks also distinguish work roles by task context, interpersonal interaction, public contact, and service or conflict-management requirements.

A workplace equity perception score was calculated from five available items: general fairness, income satisfaction, promotion satisfaction, being respected, and being able to express opinions. The items were treated as a general workplace-equity index rather than as separate subscales. Conceptually, general fairness represents an overall justice judgment, income satisfaction reflects distributive justice, promotion satisfaction reflects distributive and procedural justice, respect reflects interpersonal justice, and voice/opinion expression reflects procedural justice. The main equity index required at least three valid items out of five, so no main-model equity score was based on only one or two items. Among working respondents with industry data, 5,848 had all five equity items, 2,259 had four, 1,682 had three, and 859 had fewer than three items. Work strain was measured with fatigue, work pressure, and boredom. The item asking whether work is valuable was kept outside the work-strain index because it is a positive work-meaning item and lowered the reliability of the strain scale when forced into that construct.

Employee well-being combined standardized life happiness, life satisfaction, economic satisfaction, positive self-rated health, and low depressive symptoms. The happiness and satisfaction items anchor the composite in subjective well-being, while health and low symptoms offer a limited indicator of functioning and non-distress. The measure should therefore be read as a secondary-data well-being composite that draws on the Diener and Ryff traditions without fully measuring either framework.

### Controls

All main models adjusted for age, gender, education, non-agricultural hukou, logged income, weekly working hours, and province fixed effects. The paid-employee robustness models additionally included written labor contract and overtime status. Native missing values in these paid-employment variables were preserved as missing rather than being recoded to zero.

### Preprocessing and missing data handling

Data preprocessing used variable-specific validity rules rather than applying one global missing-code rule to every numeric variable. The analysis did not treat 96/98/99 as universal missing values. Each variable was screened according to its plausible value range and analytical role; for example, weekly working hours were retained within the 1–112-h range, and hukou was coded only from valid household-registration categories. Native missing values were preserved, labor-contract and overtime missing values were not recoded to zero, and negative or special-code income values were treated as missing before the log-income transformation because negative annual labor income is not interpretable for log-income modeling. The supplementary script writes the included-versus-excluded profile, equity-item coverage table, regression tables, bootstrap diagnostics, sensitivity outputs, and figure files used for this manuscript.

### Mathematical and analytical model

Let i denote an individual respondent and j denote industry. The industry emotional-labor requirement exposure score is represented by IELRij, the workplace equity index by Equityi, the well-being index by WBi, and the control vector by Xi. The standardized exposure variable was computed as follows:


z(IELRij)=(IELRij−mean(IELR))/SD(IELR) (1)

The statistical association model estimated the relationship between industry exposure and workplace equity perceptions:


Equityi=α0+az(IELRij)+γXi+λp+εi (2)

The outcome model estimated the direct association between exposure, equity, and well-being:


$$\mathrm{WBi}=\beta 0+c^{\prime }\;z(\text{IELRij})+b\;\text{Equityi}+\mathrm{\delta Xi}+\mathrm{\lambda p}+\mathrm{\mu i}$$ (3)

The total-association model estimated the exposure–well-being association before including workplace equity:


WBi=τ0+cz(IELRij)+θXi+λp+νi (4)

The indirect association was calculated as the product of the a and b paths:


Indirect association=a×b (5)

In these equations, lambdap represents province fixed effects and epsilon_i, mu_i, and nu_i are residual terms. Heteroskedasticity-robust standard errors were used in the main tables. As an inference check for the nested data structure, the supplementary output also reports cluster-sensitive standard errors by province and by industry for the main association models. Bootstrap confidence intervals were calculated for the indirect association in both the main and paid-employee samples using 5,000 requested draws; valid bootstrap draws are reported in the corresponding output tables.

### Ethics and legal compliance

The study uses secondary, anonymized survey data shared as a public dataset. No direct contact with participants occurred, no intervention was administered, and no personal identifiers were used in the manuscript. The original survey involved human respondents, but the present study is a secondary analysis of anonymized data; therefore, separate ethics approval for this analysis is not required by the authors’ institution unless the submitting institution requests additional confirmation. No copyrighted third-party figures were used; all figures in this manuscript were newly produced from the analytical outputs.

The analytical workflow is summarized in Figure 2, while Table 2 defines the measures used in the models and Table 3 reports the final sample construction.

**Figure 2:**
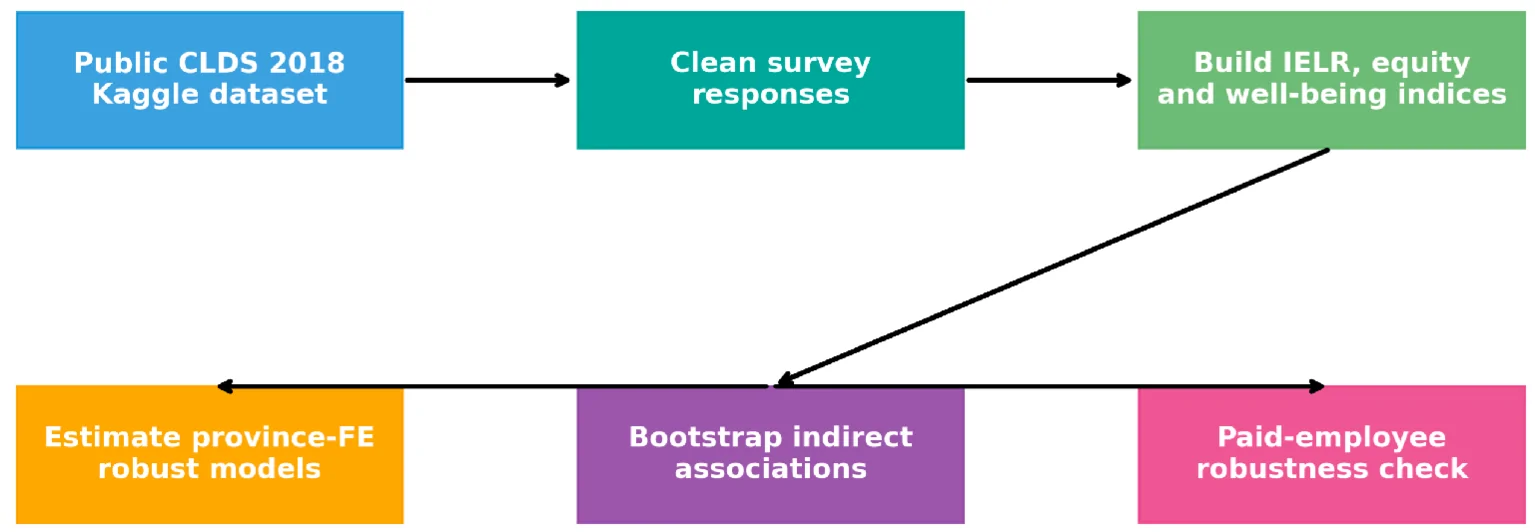
Data preparation and analysis workflow for the corrected CLDS 2018 working-sample analysis. Complete - case main sample: *N* = 9,361 | paid employee robustness sample: *N* = 1,963.

**Table 2:** Dataset variables and operationalization.

Construct/variable	Analytical role	Operational definition	Interpretation
Industry-level emotional-labor exposure proxy (IELR)	Independent exposure proxy	Sector-level score from 1 = low to 5 = high expected public-facing emotional requirement	Industry category proxy; not individual emotional-labor behavior
Workplace equity perception	Statistical indirect-association variable	Mean of fairness, income satisfaction, promotion satisfaction, respect, and voice/opinion expression; at least 3 of 5 valid items required	Higher = stronger equity perception
Employee well-being	Dependent variable	Mean of standardized life happiness, life satisfaction, economic satisfaction, positive health, and low depressive symptoms	Higher = better well-being
Psychological distress	Supplementary outcome	Mean of 20 depressive-symptom frequency items	Higher = worse distress
Work strain	Supplementary outcome	Mean of fatigue, work pressure, and boredom items	Higher = worse strain
Controls	Covariates	Age, gender, education, non-agricultural hukou, logged income, weekly hours, province fixed effects; paid sample also includes written contract and overtime	Included in regression models

IELR is an industry-level exposure proxy because the dataset does not contain a direct individual emotional-labor scale. Workplace equity requires at least three valid items out of five in the main analysis.

**Table 3:** Sample construction summary.

Sample construction statistic	Value
Total observations in IID.dta	16,537
Working respondents with industry data	10,648
Main complete-case sample	9,361
Paid employee subsample with industry data	2,348
Paid employee complete-case robustness sample	1,963

The sample is a common complete analytical sample across the main and supplementary outcome models; it is not described as a mediation-only complete-case sample.

The paid-employee subsample is used as a robustness check. Missing values in written-contract and overtime variables are preserved as missing, not recoded to zero.

Table 2 lists the operational variables used to convert the CLDS 2018 items into the analytical constructs for this article.

Table 3 documents the movement from the full CLDS 2018 file to the main complete-case sample and the paid-employee robustness sample (Tables 4, 5).

**Table 4:** Equity-item coverage and complete-case diagnostic.

Equity items available	*N*	Percent of working respondents with industry data
0	4	0.0%
1	90	0.8%
2	765	7.2%
3	1,682	15.8%
4	2,259	21.2%
5	5,848	54.9%

The final main equity index requires at least three valid equity items. Respondents with one or two equity items are therefore excluded from the main complete-case equity-index calculation.

**Table 5:** Included-versus-excluded diagnostic for working respondents with industry data.

Variable	Included N	Included mean	Excluded N	Excluded mean	Difference
Age	9,361	46.806	1,252	52.678	−5.872
Female	9,361	0.455	1,287	0.490	−0.035
Education	9,361	3.689	1,274	2.547	1.142
Logged income	9,361	9.551	1,283	8.894	0.658
Weekly working hours	9,361	44.424	850	39.925	4.499
IELR exposure	9,361	2.258	1,287	1.548	0.710
Equity, all available items	9,361	3.314	1,283	3.105	0.209
Well-being index	9,361	0.001	1,283	−0.097	0.098

The comparison uses working respondents with usable industry information. The complete analytical sample retained 9,361 of 10,648 respondents (87.9%). Complete-case analysis was used for transparency and direct reproducibility. Multiple imputation was not adopted because the public-use CLDS 2018 file available for this analysis does not provide enough auxiliary information or item-specific missingness documentation to support a confident missing-at-random assumption for the workplace-equity non-response pattern. The diagnostic does not remove the possibility of selection bias; it shows the direction and magnitude of included-versus-excluded differences and supports treating the estimates as reproducible complete-case associations rather than population estimates.

## Results

### Descriptive statistics and construct reliability

Table 6 reports descriptive statistics for the corrected main analytical sample. The mean IELR score was 2.258, indicating that the complete-case sample remained concentrated in lower-to-moderate exposure industries. The mean workplace equity index was 3.314 on a 1 to 5 scale. The well-being index was centered close to zero, while the mean CES-D distress score was 1.384 and the mean three-item work-strain score was 2.606. Figure 3 visualizes the updated distributions of the three central constructs.

**Table 6:** Descriptive statistics for the main analytical variables.

Variable	Count	Mean	Std	Min	Max
IELR exposure	9,361	2.258	1.590	1.000	5.000
Workplace equity	9,361	3.314	0.645	1.000	5.000
Well-being	9,361	0.001	0.695	−3.345	1.344
Psychological distress	9,361	1.384	0.452	1.000	4.000
Work strain	9,361	2.606	1.110	1.000	5.000
Age	9,361	46.806	12.775	15.000	80.000
Female	9,361	0.455	0.498	0.000	1.000
Education	9,361	3.689	2.428	1.000	11.000
Logged income	9,361	9.551	2.405	0.000	14.914
Weekly working hours	9,361	44.424	21.524	1.000	112.000

Well-being is a standardized composite. Female is coded as a binary indicator in the analytical dataset.

**Figure 3:**
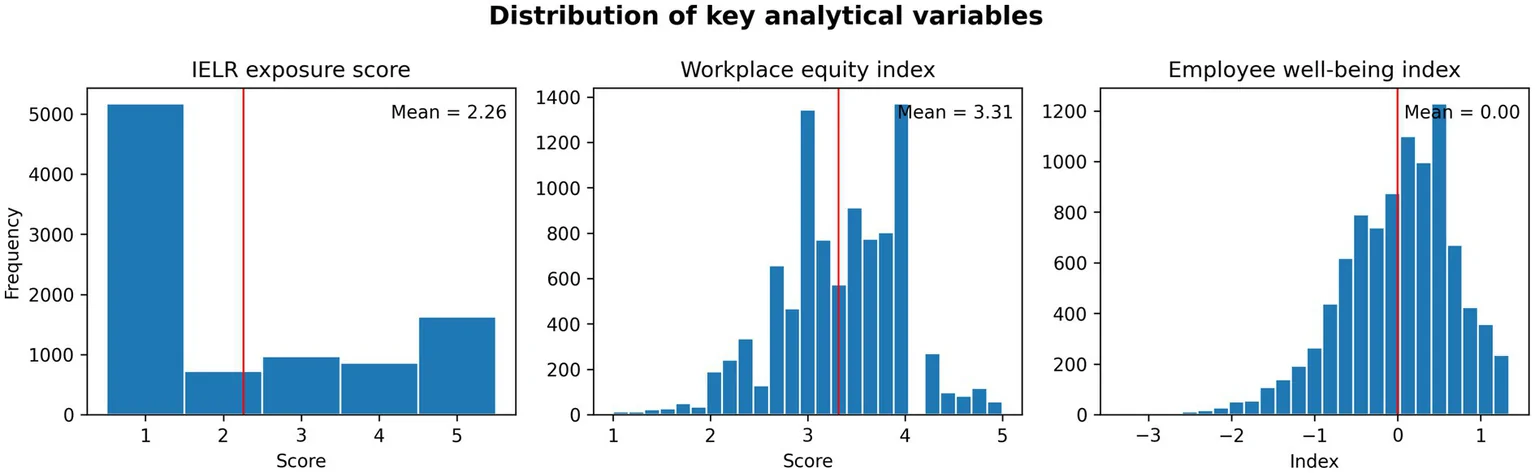
Distributions of industry-level emotional-labor exposure proxy, workplace equity, and employee well-being in the corrected main sample.

Reliability estimates were calculated using item-complete cases for each respective scale within the main analytical sample. For the five-item workplace-equity/justice-fairness scale, Cronbach’s alpha was based on respondents with complete responses to all five fairness items (*N* = 5,631), whereas the regression sample remained *N* = 9,361 because the main equity index required at least three valid equity items. The alpha values were: workplace equity/justice-fairness = 0.786 (*N* = 5,631), employee well-being = 0.744, psychological distress = 0.947, and the three-item work-strain scale = 0.768. These values support the retained composite indicators for the analysis, while the IELR score remains an industry-level exposure proxy rather than a psychometric scale.

### Correlations

Table 7 and Figure 4 report the corrected correlation structure. IELR had a small positive correlation with workplace equity (r = 0.076) and well-being (r = 0.147), and small negative correlations with distress (r = −0.092) and three-item work strain (r = −0.139). Workplace equity showed a strong positive correlation with well-being (r = 0.480) and negative correlations with distress (r = −0.239) and three-item work strain (r = −0.310).

**Table 7:** Pearson correlation matrix for the analytical constructs.

Variable	IELR	Equity	Well-being	Distress	Work strain
IELR	1.000	0.076	0.147	−0.092	−0.139
Equity	0.076	1.000	0.480	−0.239	−0.310
Well-being	0.147	0.480	1.000	−0.606	−0.366
Distress	−0.092	−0.239	−0.606	1.000	0.345
Work strain	−0.139	−0.310	−0.366	0.345	1.000

Positive correlations indicate that two variables move together. Higher distress and work strain indicate worse outcomes.

**Figure 4:**
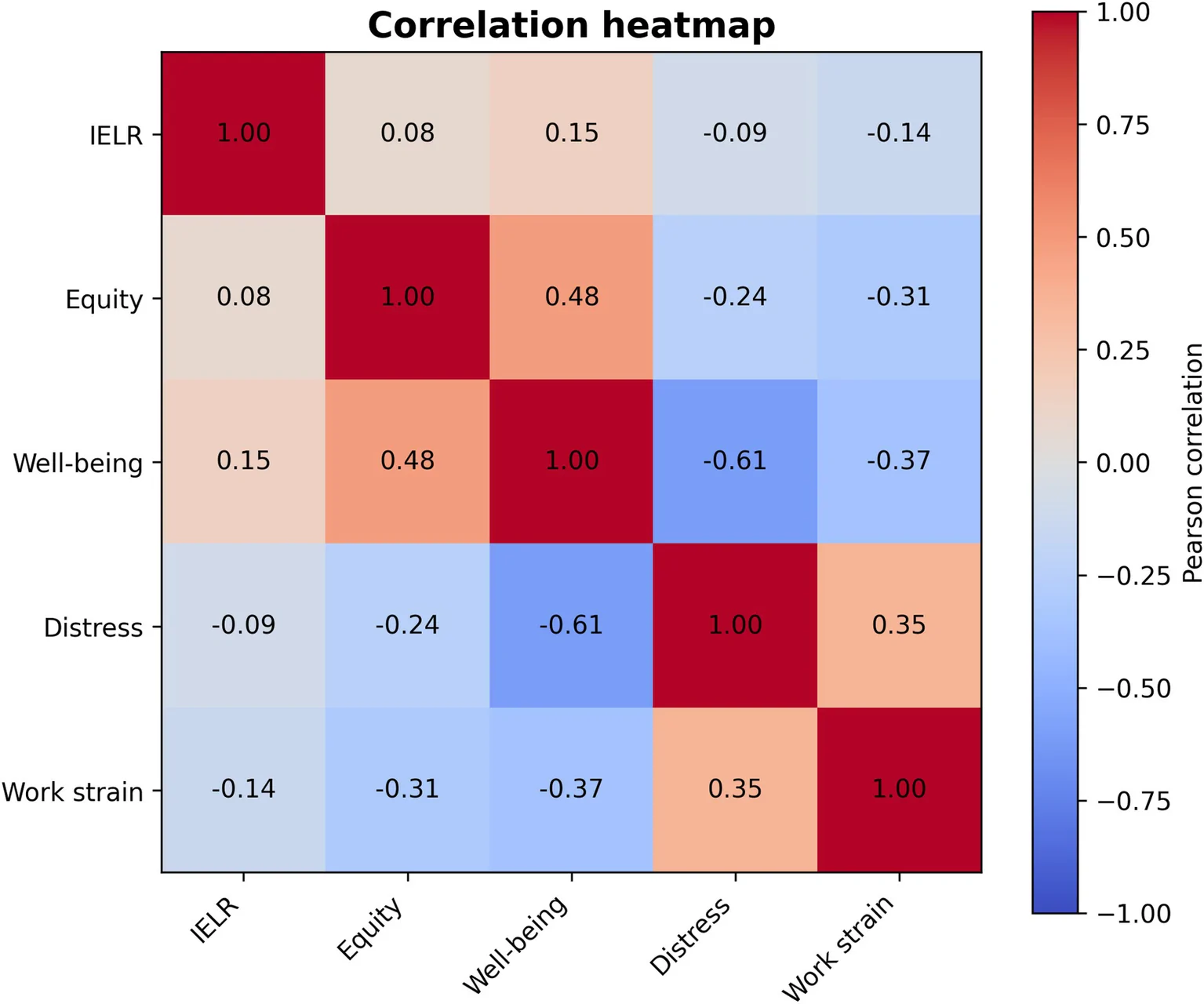
Correlation heatmap for IELR, workplace equity, well-being, distress, and work strain in the corrected main sample.

### Industry exposure profile

Industry profiles differed across the coded IELR categories. Wholesale/retail trade and catering, social services, and health/sport/social welfare were assigned the highest IELR scores because their modal work tasks involve frequent service, care, or public-facing interaction. Agriculture, mining, and manufacturing were assigned lower IELR scores because their modal work tasks involve less direct emotional display toward clients or the public. As shown in Figure 5, higher IELR categories tended to report higher workplace equity and slightly higher average well-being, while the corrected three-item work-strain profile is interpreted descriptively rather than causally.

**Figure 5:**
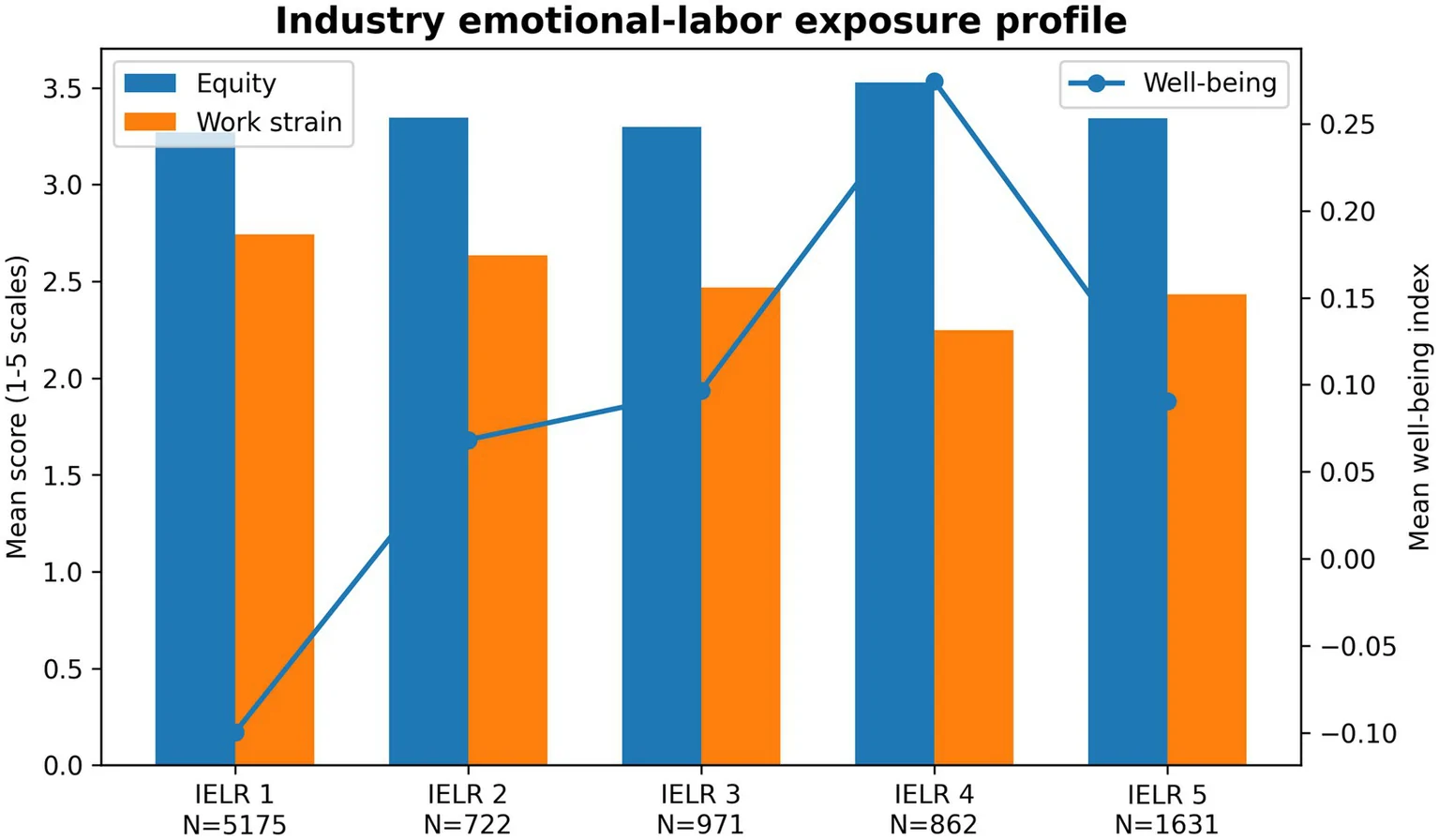
Industry exposure profile showing mean workplace equity, work strain, and well-being across IELR exposure categories in the corrected main sample.

### Regression findings

Table 8 reports the province-fixed-effect regression results for the main working sample. IELR was positively associated with workplace equity perceptions (beta = 0.063, *p* < 0.001), supporting H2 as a statistical association. The total association between IELR and well-being was positive but small (beta = 0.067, *p* < 0.001), supporting H1 as an association. When workplace equity was included in the outcome model, the direct IELR association remained positive but small (beta = 0.038, *p* < 0.001), while workplace equity showed a much stronger association with well-being (beta = 0.460, *p* < 0.001), supporting H3. All coefficients and R2 values in Table 8 come from the same province-fixed-effect specification and the same *N* = 9,361 analytical sample.

**Table 8:** Main working-sample regression results.

Model	Term	Beta	Robust_SE	p_value	*N*	R^2^
M1 Equity association	IELR (z)	0.063	0.011	<0.001	9,361	0.074
M2 Well-being	IELR (z)	0.038	0.010	<0.001	9,361	0.295
M2 Well-being	Workplace equity (z)	0.460	0.010	<0.001	9,361	0.295
M3 Total association	IELR (z)	0.067	0.011	<0.001	9,361	0.100
M4 Distress	IELR (z)	−0.037	0.012	0.001	9,361	0.107
M4 Distress	Workplace equity (z)	−0.234	0.012	<0.001	9,361	0.107
M5 Work strain	IELR (z)	−0.068	0.011	<0.001	9,361	0.161
M5 Work strain	Workplace equity (z)	−0.301	0.010	<0.001	9,361	0.161

Heteroskedasticity-robust standard errors are reported. Province fixed effects and controls are included in all models.

Workplace equity was also negatively associated with psychological distress (β = −0.234, *p* < 0.001) and three-item work strain (β = −0.301, *p* < 0.001). These findings support H5 and show that workplace equity is a more stable correlate of employee well-being and strain than the industry-level exposure proxy.

### Statistical indirect association analysis

The statistical indirect-association results are summarized in Table 9 and Figure 6. The estimated main-sample indirect association between IELR and well-being through workplace equity was 0.029, with a 95% bootstrap confidence interval from 0.019 to 0.039 based on 5,000 valid bootstrap draws. Because the total IELR association is small, the proportion represented by the indirect association should be interpreted cautiously. The substantive pattern is that workplace equity is the stronger and more consistent correlate of well-being.

**Table 9:** Main working-sample statistical indirect-association estimates.

Path	Estimate
a: IELR → Equity	0.063
b: Equity → Well-being	0.460
c: Total IELR → Well-being	0.067
c-prime: Direct IELR → Well-being	0.038
Indirect association a*b	0.029
Bootstrap CI lower	0.019
Bootstrap CI upper	0.039
Proportion represented by indirect association	0.432

Bootstrap confidence interval refers to the indirect association a × b.

**Figure 6:**
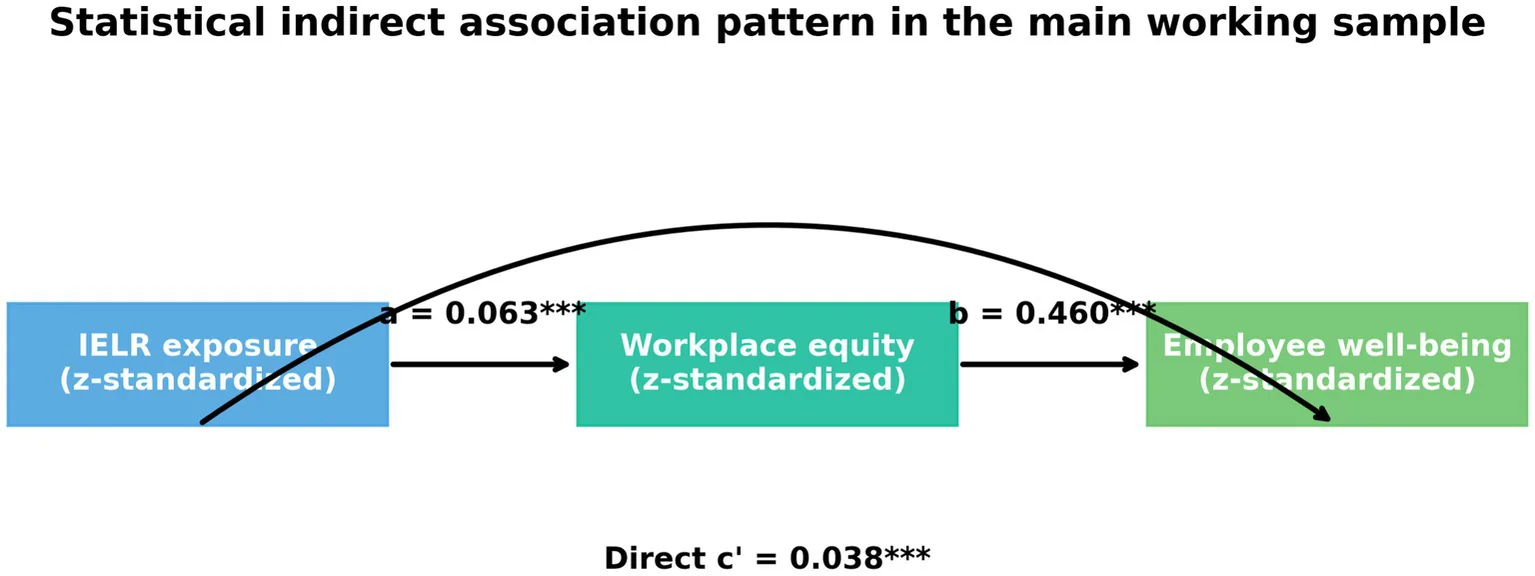
Statistical indirect-association diagram for the main working sample with standardized coefficients. Indirect association = 0.029; 95% bootstrap CI [0.019, 0.039].

Figure 7 summarizes the key standardized regression effects from the main models and shows the same pattern visually: the workplace-equity coefficient is much larger than the IELR coefficients, while equity is negatively associated with distress and three-item work strain.

**Figure 7:**
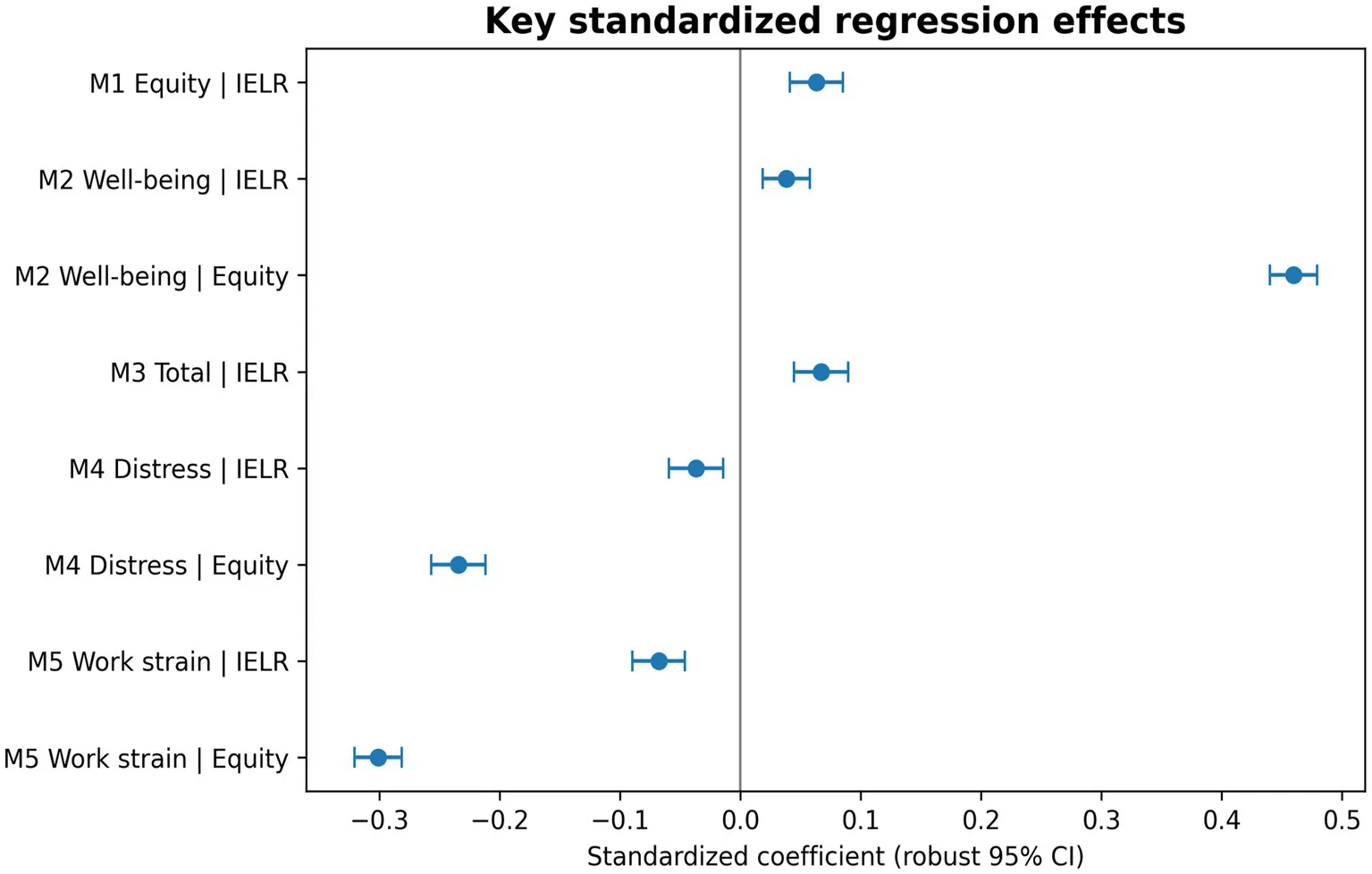
Key standardized regression effects in the corrected main working sample, with 95% confidence intervals.

### Robustness check in paid employees

Table 10 reports the paid-employee robustness sample. After preserving missing values in labor-contract and overtime variables, the complete-case paid-employee sample included 1,963 respondents. The IELR-to-equity association was not statistically detectable in this restricted sample (β = 0.023, *p* = 0.343), and the direct IELR-well-being association was also not statistically detectable (β = 0.016, *p* = 0.463). Workplace equity remained strongly associated with well-being (β = 0.448, *p* < 0.001) and was also negatively associated with three-item work strain (β = −0.301, *p* < 0.001). The paid-employee indirect association was small (0.010), and the 95% bootstrap interval crossed zero (−0.010 to 0.031; 4,949 valid resamples from 5,000 requested draws), so the paid-employee analysis does not provide strong support for the indirect pattern.

**Table 10:** Paid-employee robustness regression results.

Model	Term	Beta	Robust_SE	p_value	*N*	R^2^
M1 Equity association	IELR (z)	0.023	0.024	0.343	1,963	0.072
M2 Well-being	IELR (z)	0.016	0.022	0.463	1,963	0.270
M2 Well-being	Workplace equity (z)	0.448	0.021	<0.001	1,963	0.270
M3 Total association	IELR (z)	0.026	0.024	0.287	1,963	0.083
M4 Distress	IELR (z)	−0.005	0.025	0.832	1,963	0.084
M4 Distress	Workplace equity (z)	−0.210	0.024	<0.001	1,963	0.084
M5 Work strain	IELR (z)	−0.038	0.023	0.105	1,963	0.150
M5 Work strain	Workplace equity (z)	−0.301	0.023	<0.001	1,963	0.150

The robustness sample retains only paid employees with complete analytical data after native missing values in contract and overtime variables are preserved. The direct IELR–well-being association is not statistically detectable in this restricted sample.

Additional diagnostic checks are reported in the supplementary output and support the same cautious interpretation. Removing logged income did not alter the pattern: IELR remained small and positive in the well-being model (beta = 0.043), while workplace equity remained much stronger (beta = 0.463). Using a positive-only well-being index gave a smaller IELR coefficient (beta = 0.032) and a stable equity coefficient (beta = 0.458). Alternative IELR mappings for mixed industries produced direct IELR coefficients between 0.034 and 0.041, and cluster-sensitive standard errors by province and industry did not change the conclusion that the equity association is the dominant empirical pattern. These checks are not treated as new main models; they are included to show that the central interpretation is not dependent on a single coding choice (Table 11).

**Table 11:** Paid-employee bootstrap statistical indirect-association estimates.

Path	Estimate
a: IELR → Equity	0.023
b: Equity → Well-being	0.448
c: Total IELR → Well-being	0.026
c-prime: Direct IELR → Well-being	0.016
Indirect association a*b	0.010
Bootstrap CI lower	−0.010
Bootstrap CI upper	0.031
Valid bootstrap draws used	4,949
Proportion represented by indirect association	0.389

Bootstrap confidence interval refers to the paid-employee indirect association a x b. The script requested 5,000 resamples; 4,949 valid resamples were retained after singular resamples were skipped.

## Discussion

### Summary of key findings

IELR was found to be positively associated with well-being overall, rather than negatively associated, in this corrected CLDS 2018 analysis. The stability of findings is that workplace equity is strongly linked to increased well-being and reduced work strain (measured by the 3 items) and distress, while the industry-level exposure proxy is only weakly associated and is not statistically detectable in the paid-employee robustness sample.

It is important to note that the IELR correlation is positive, but this does not mean that emotional labor contributes to well-being. Higher-scoring industries may also contain more formal service, public, education, health, finance and welfare types of industries, where employees have higher perceptions of equity in their jobs. The IELR coefficient can be small when equity is added on. Here, therefore, the central interpretation is somewhat more cautious: equity seems to take on more substantive meaning than does the industry exposure score.

### Theoretical implications

The first theoretical implication is of the need to analyse emotional labor at more than one level. Emotional labor strategies at the level of the individual are still relevant, and sector and occupation can influence the emotional demands. The study presented here shows a pragmatic approach to analyzing industry-level emotional-labor exposure without having direct measures of emotional labor surface acting and deep acting in a national labor force data source.

The second implication is that workplace equity can be considered as a valuable resource for work. The statistical model places equity between industry exposure and well-being, however, due to the cross-sectional design, this should be interpreted as an indirect-association model, not as evidence of a causal mechanism. The take-home message remains: Fairness, respect and voice are more consistently associated with well-being than the general exposure to the industry proxy.

The third implication is that employee well-being should not be equated with the absence of distress. The equity index was associated with the positive well-being composite and was also associated with lower psychological distress and lower three-item work strain. This twofold pattern suggests that fair work experiences may be linked to both stronger positive functioning and lower negative work-related load.

### Practical implications

The results suggest that for employers in industries that require high levels of emotion, equity practices are more tangible than symbolic. Wellness slogans might be just as important as fair pay systems, transparent promotion channels, respectful supervision and authentic voice channels. People who regularly deal with customers and/or public emotions should have evidence that the company is aware of the skill and strain involved for the worker. Managers should also consider listening to employees as a “watchdog. Emotionally challenging tasks can lead to unexpressed frustration, which may cost you more than it helps you earn. Voice channels provide an opportunity for workers to notice and share: unfair treatment, customer aggression, unrealistic display rules, or workload pressure, before they become: burnout, withdrawal, or turnover issues.

The study recommends that both demand- and equity-based indicators can be used to assess the emotionally demanding industries for policymakers and labor researchers. Emotional labor is not just a psychological problem – it is associated with decent work, rewards fairness, health and dignity in the industry. It is thus recommended to include direct emotional-labor scales in future waves of the labor survey together with scales of justice, voice and well-being.

### Limitations

The primary constraint is measurement. The individual scales of emotional-labor, namely surface acting, deep acting, emotional dissonance and display rules, are not included in CLDS 2018. IELR is an exploratory industry-level exposure proxy and should be validated against occupational demand proxies such as O*NET interpersonal/task descriptors or occupation measures linked to ISCO-08, if available, for future studies. The positive work-value item was excluded from the work strain measure, which included fatigue, work pressure and boredom, as it decreased the reliability of the measure when included in the strain construct.

Secondly, there is methodological identification and reporting bias. This may be due to common method bias as the same survey environment was used to assess workplace equity, well-being, distress, and strain. Common-source bias is also a concern since the same respondents provided the perceptions on the predictor side as well as the evaluations on the outcome side. Associations can be inflated or distorted due to self-report bias when those with higher positive or negative response styles rate multiple constructs in the same direction. The reverse causality is a possibility as well, since employees who are better off may also rate workplace fairness as more favorable than others. Industry assignment could also be endogenous because well-being can be influenced by industry location, and local labor market characteristics, family resources or family health status unobserved by researchers can affect industry location. Self-selection bias may arise from when workers join, stay in or exit an industry due to factors that are not included in the dataset and are linked to health, income security, education or emotional demands. The inclusion of province fixed-effects, observed controls, paid-employee checks and cluster-sensitive diagnostics alleviates some concerns but cannot overcome the potential for these sources of bias.

A third limitation concerns survey design and missing data. The analysis does not apply CLDS survey weights and does not fully model household or community clustering, so the estimates should not be presented as weighted population estimates. Complete-case selection also leaves a non-random included group, as shown by the included-versus-excluded diagnostic. Multiple imputation could be considered in future work if richer auxiliary variables and item-specific missingness documentation are available. These design limits do not invalidate the reported complete-case associations, but they narrow the scope of inference.

### Future research

Future studies need to integrate the industry-level exposures with the individual emotional-labor scales to disentangle the industry requirements from the individual regulatory strategies. A better design would follow employees over time and determine whether changes in any of the measures of pay justice, promotion justice, supervisor respect, or voice are correlated with changes in measures of well-being. Other factors to consider as moderators include ethical leadership, decent work practices, workplace spirituality, gender and employment form. Qualitative work would also be helpful. Survey coefficients indicate that equity is a concern, but interviews can provide meaning when workers are asked to be happy, caring, calming, and able to absorb frustration and/or anger. A more holistic view of emotional labor and dignity at work could be achieved if national data were linked to workers’ narratives.

Future work should also test the same framework with direct emotional-labor scales, occupationally coded exposure measures, survey weights, household/community clustering, and missing-data approaches such as multiple imputation. These additions would help separate industry exposure from individual job tasks and reduce the uncertainty created by complete-case selection. In particular, any later multiple-imputation approach should justify the missing-at-random assumption with auxiliary variables and item-level missingness evidence rather than using imputation only to increase sample size.

## Conclusion

The present study employed CLDS 2018 to test for statistical consistency in perceptions of equity in the workplace and an indirect relationship between an industry-level proxy for exposure to emotional labor and employee well-being. The corrected analysis revealed a small positive association between IELR and well-being in the overall working sample, but no statistically significant direct association between IELR and well-being among paid employees, and consistently strong association between workplace equity and well-being. There was also a consistent association between workplace equity and lower psychological distress and lower three item work strain. The main finding is thus tentative, pragmatic and optimistic: fair, respectful and voice-enabling workplaces are better associated with the employee well-being than is the industry exposure proxy used in this exploratory study.

## Data Availability

The original contributions presented in the study are included in the article/supplementary material, further inquiries can be directed to the corresponding author.
